# Persistent nonequilibrium dynamics of the thermal energies in the spin and phonon systems of an antiferromagnet

**DOI:** 10.1063/1.4961253

**Published:** 2016-09-07

**Authors:** A. von Reppert, J. Pudell, A. Koc, M. Reinhardt, W. Leitenberger, K. Dumesnil, F. Zamponi, M. Bargheer

**Affiliations:** 1 Institut für Physik and Astronomie, Universität Potsdam, Karl-Liebknecht-Str. 24-25, 14476 Potsdam, Germany; 2 Helmholtz Zentrum Berlin, Albert-Einstein-Str. 15, 12489 Berlin, Germany; 3 Institut Jean Lamour (UMR CNRS 7198), Université Lorraine, Boulevard des Aiguillettes B.P. 239, F-54500 Vandoeuvre les Nancy Cédex, France

## Abstract

We present a temperature and fluence dependent Ultrafast X-Ray Diffraction study of a laser-heated antiferromagnetic dysprosium thin film. The loss of antiferromagnetic order is evidenced by a pronounced lattice contraction. We devise a method to determine the energy flow between the phonon and spin system from calibrated Bragg peak positions in thermal equilibrium. Reestablishing the magnetic order is much slower than the cooling of the lattice, especially around the Néel temperature. Despite the pronounced magnetostriction, the transfer of energy from the spin system to the phonons in Dy is slow after the spin-order is lost.

## INTRODUCTION

The unexpectedly fast magnetization loss in magnetic thin films upon photoexcitation observed two decades ago^1^ stimulated extensive research aiming at ultrafast data storage and related applications. Experiments using different schemes for probing the changes induced in magnetic systems by optical light pulses have been employed. They range from visible magneto-optical Kerr effect (MOKE),^2,3^ extreme ultraviolet MOKE,^4^ x-ray magnetic circular dichroism^5–8^ over photoelectron spectroscopy^9–11^ to ultrafast x-ray diffraction (UXRD)^12^ and resonant x-ray scattering.^13^ The discussions are mainly focused on the transfer of angular momentum or the loss of spin-order in the framework of two- or three-temperature models. Antiferromagnets are believed to support even faster local angular momentum changes, since the average spin remains zero.^7,14^ The multifarious behavior of transition metals and 4f magnetic materials, however, have eluded so far the effort to be described by a unique physical picture.^3,15,16^ The demagnetization aspects at ultrashort timescales are an exciting first step of the full magnetization dynamics. The remagnetization is another fundamental issue, especially in the perspective of possible technological applications, which has received less attention. Few authors have approached the problem of how fast and through which intermediate steps the remagnetization occurs.^13,17–20^ Different models have been developed to simulate the dynamics on longer timescales.^21–24^ Some of them point out the important role of fluctuations and domain formation close to the phase transition. In particular, resonant x-ray diffraction experiments from the dysprosium spin-spiral have shown that some disorder of the spin system persists more than 10 ns, especially for high fluence excitation.^13^

However, a characterization of the influence of the temperature and excitation density on the remagnetization dynamics is still missing. In fact, a detailed account of the energy in the phonon and spin systems at the different stages of the remagnetization has not been reported. Dysprosium (Dy) is a thoroughly characterized material^25–28^ with a pronounced magnetostrictive expansion of the lattice constant *c*. The 4f electrons, coupled via the 5d electrons (i.e., RKKY coupling), are responsible for its magnetic properties.^27,28^ All magnetic materials show magnetostriction, i.e., a change of the lattice constant that depends on the magnetization *M*(*T*). Only a single UXRD study has so far explored the magnetostrictive strain on ultrafast timescales,^12^ although the existing UXRD setups have been constantly improved and allow for measuring minute lattice strains on ultrafast timescales.^29^ In general, the lattice strain occurs as a response to stress imposed by a modified energy density. This is particularly evident, when the Grüneisen concept is used, e.g., for separating the electron and phonon contribution to photoinduced stress.^30,31^ Although the extension to magnetic systems was suggested,^30^ this powerful approach has not been realized.

Here, we analyze temperature- and excitation-fluence-dependent UXRD data from an 80 nm Dy thin film to characterize the nonequilibrium spin and lattice dynamics triggered by ultrafast heating of the electron system by a near-infrared laser pulse. The central and surprising result of our analysis can be directly read from the experimental data: The lattice contraction originating from the spin-disorder persists on the nanosecond timescale when the lattice heat is already dissipated to the substrate. The Bragg peak shift is a direct measure of the time-dependent strain ε(t)=Δc(t)/c in the out-of-plane lattice constant *c* which is linearly related to the deposited heat $Q_{\text{tot}}$ in the film. From these two measured variables ϵ(t) and *Q_tot_*, we determine the transient energy densities $Q_{P}(t)$ and $Q_{S}(t)$ in the phonon and spin excitations, respectively, from a numerically robust analysis. Our model adopts the linear relation of these energy densities to the experimentally observed lattice strain via separate nearly temperature independent constants for the phonon and spin systems (see Eq. (1)). Therefore, our analysis in a “two-thermal-energies-model” (TTEM) is independent of modifications of the spatial excitation profile. In comparison with a “two-temperatures-model” (TTM),^1,32^ our TTEM has the advantage that the energy contained in random motion of the phonon and spin excitations is a well-defined quantity even in strong non-equilibrium-situations, where the temperature is not.

The electron system of the Y cap layer and the dysprosium layer are photoexcited, and the multilayer system is cooled through the substrate. Here, we examine how the thermal energy in the dysprosium layer is distributed between the phonon and the spin system.

## RESULTS

### Linear relation of strain to lattice- and spin-energy

The sample is an 80 nm thick (0 0 0 1) oriented Dy film grown on a sapphire substrate with a 100 nm Niobium and 10 nm Yttrium (Y) buffer layer. The structure is capped with another 18 nm thin Y layer (see Fig. 3(e)) in order to support the helical spin ordering in the thin Dy film^34^ and to prevent oxidation.

**FIG. 1.**
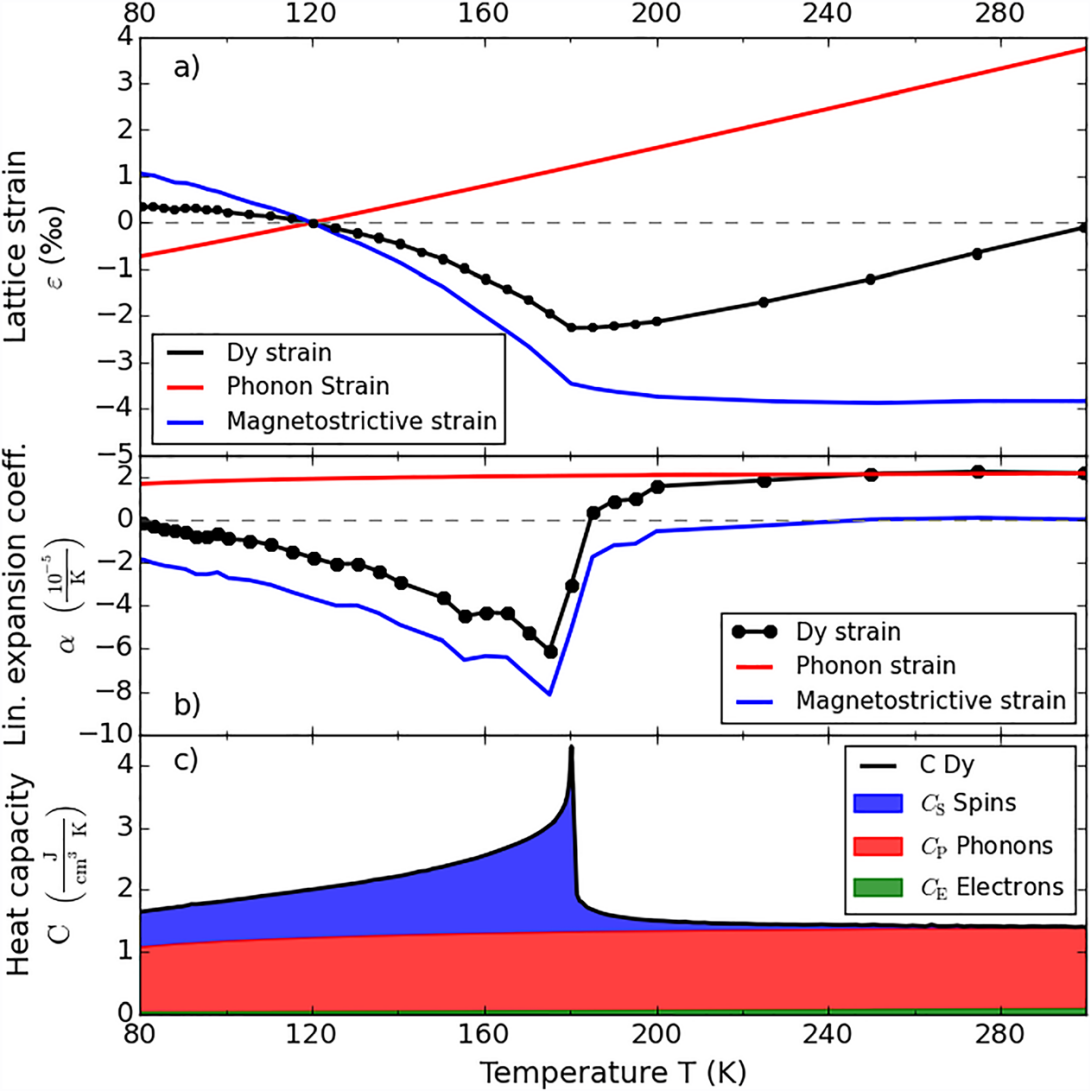
Temperature dependence of the equilibrium properties of Dy. (a) Black: strain along the *c*-axis in the Dy film obtained by static X-Ray diffraction measurements on the hcp (0 0 0 2) reflection. The red and blue curves represent the lattice strain contributions for heating the phonons and the magnetic system. (b) Thermal expansion coefficients of Dy derived from the data in panel (a). (c) Heat capacity reported for bulk Dy^33^ decomposed into $C_{P},\;C_{S}$, and $C_{E}$.

We have carefully measured the lattice constant *c*(*T*) of the Dy thin film as a function of temperature. The lattice strain derived from the Bragg peak position (black dots in Fig. 1(a)) shows a pronounced expansion below the Néel temperature $T_{N}=180\;K$, which results from the antiferromagnetic spin-ordering. Fig. 1(b) shows the linear expansion coefficient α(T) derived from this measurement, with a pronounced negative thermal expansion below 180 K. The constant-pressure specific heat *C* in Fig. 1(c) is taken from the literature (black line).^33^ We separated the strain $\varepsilon (T)=\varepsilon_{P}(T)+\varepsilon_{S}(T)$, its derivative α(T), and the heat capacity $C(T)=C_{P}(T)+C_{S}(T)$ into phonon and spin contributions (cf. Fig. 1). We determined the combined electron and phonon contribution from the specific heat of the non-magnetic rare earth lutetium,^35^ which is in agreement with a simple Debye approximation. Near its maximum at *T_N_* the spins carry more than half of the specific heat, whereas the specific heat of the electrons is negligible in the relevant temperature range. Fig. 1(a) exemplifies the separation of the strain $\varepsilon (T)=\varepsilon_{P}(T)+\varepsilon_{S}(T)$ for the initial temperature *T*_i_ = 120 K. This is the equilibrium temperature given by the thermostate before deposition of the heat *Q* by the laser pulse.

**FIG. 2.**
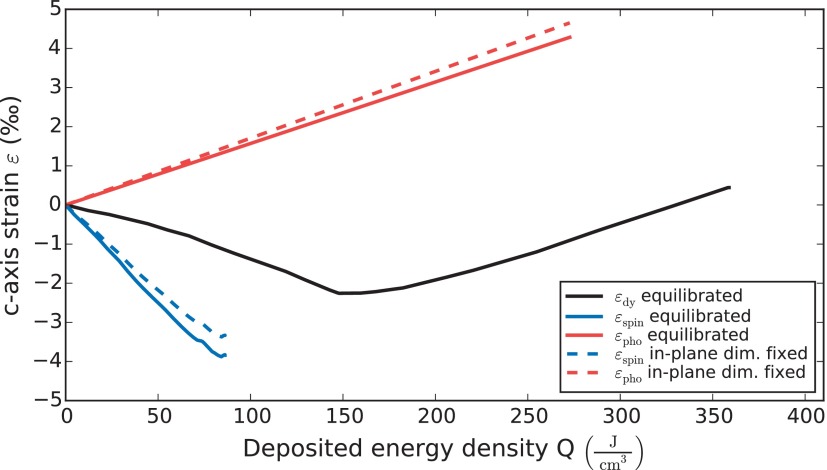
Lattice strain as a function of the total heat *Q_tot_* (black), for *T*_Start_ = 120 K. The red and blue curves show the separation into strain driven by heat in the phonons *Q_P_* and in spin excitations *Q_S_*. Although the black line is strongly curved, the separation into spin and phonon contributions yields a linear dependence. Note that the maximum heat in the spin system is given by the blue area in Fig. 1(c), i.e., the spin contribution to the specific heat *C_S_*. The dashed lines show the result after taking into account that, on ultrafast timescales, the lattice cannot expand in plane.

In thermal equilibrium, more and more modes with frequency *ω* fulfill the relation $k_{B}T>\hbar \omega$ with rising temperature, which therefore contribute a thermally accessible degree of freedom.^36^ As a consequence, both the thermal expansion coefficient *α* and the specific heat strongly vary with the temperature *T*. However, if we numerically calculate the lattice strains $\varepsilon_{P,S}$ as a function of the energy densities $Q_{P,S}$, a linear relation is obtained (see solid lines in Fig. 2). While the phonon contribution simply extrapolates to high *Q* until the melting of the lattice is observed, the spin contribution to the energy density is limited (blue area in Fig. 1(c)). The linear relation of strain and energy suggests the definition of parameters $\beta_{P,S}=C_{P,S}(T)/\alpha_{P,S}(T)$ which measure the infinitesimal energy density required to yield an additional strain at the temperature *T*. These parameters are essentially inverse macroscopic Grüneisen constants $\Gamma_{P,S}=K/\beta_{P,S}$, where *K* is the bulk modulus of Dy.^37^

Microscopic theories define mode-specific Grüneisen constants, which may vary both in magnitude and sign.^38^ However, if we assume a non-equilibrium situation which has no selective population of special modes, we can assume the lattice strain

$$\varepsilon_{P,S}=\int_{T}^{T+\Delta T}\alpha_{P,S}(T)dT=\frac{Q_{P,S}}{\beta_{P,S}}=\frac{\Gamma_{P,S}Q_{P,S}}{K}$$ (1)

to be proportional to the average increase of the energy density $Q_{P}$ and $Q_{S}$ of the phonon and spin systems, respectively. The parameters *β* or Γ are independent of *T* to a first approximation, making our non-equilibrium data analysis very robust. While $\Gamma_{P}\approx 0.6$ and $\Gamma_{S}\approx -1.8$ are dimensionless quantities, *β_P_* = 63 kJ/cm^3^ and *β_S_* = –20 kJ/cm^3^ can be interpreted as a characteristic energy densities required to expand or contract a solid. For example, the energy density Q=0.01·β expands or contracts the solid by 1%, depending on the sign of *β*. The weak temperature dependence of the bulk modulus *K* ≈ 41 GPa* *= 41 kJ/cm^3^ adds some subtle difference near the phase transition. We note that for the same amount of energy the lattice contraction resulting from the spin excitation is three times larger than the expansion due to phonon heating, also above $T_{N}$. Even above $T_{N}$, where the spin-contribution to the specific heat is strongly reduced (Fig. 1(c)), the negative thermal expansion driven by spin excitations persists (Fig. 1(b)).

### Ultrafast x-ray diffraction data

**FIG. 3.**
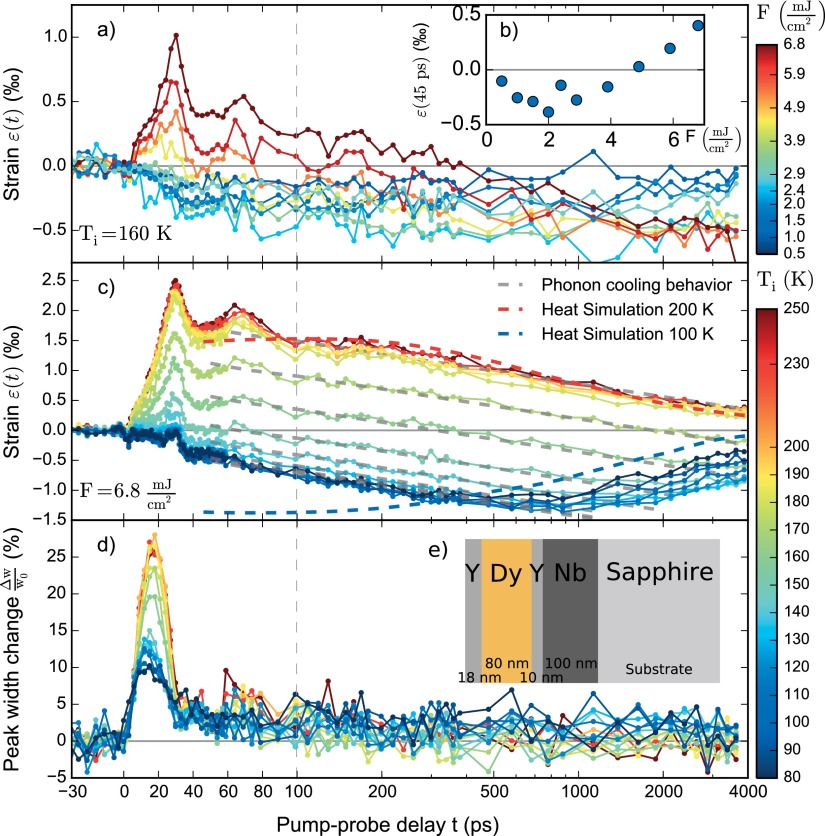
UXRD data. (a) Time resolved lattice strain ε(t) for $T_{i}=160\;K$ for different excitation fluences. (b) ε(45 ps) as a function of fluence. The initial strain rises linearly above the threshold fluence *F*_th_ = 2 mJ/cm^2^. (c) Lattice strain ε(t) and (d) the relative peak width change for different initial temperatures at fixed excitation fluence *F* = 6.8 mJ/cm^2^. Dashed lines in (c) indicate the expected lattice strain according to simulations of the heat conduction. (e) Layering sequence of the sample.

We now describe the UXRD data of Dy after ultrafast heating of the electrons in Y and Dy by a 100 fs, 800 nm laser pulse. The optical penetration depth of 24 nm was determined by ellipsometry. The lattice response is probed by diffraction of 250 fs Cu-K_*α*_ pulses originating from a tabletop Plasma-X-Ray source.^29,39^ Fig. 3(a) shows the transient strain ε(t) for different pump fluences *F* of the excitation pulse at the initial temperature $T_{i}=160\;K<T_{N}$. The incident fluence *F* is determined as the average intensity divided by the pumped area on the sample in the top-hat approximation, and the pump pulse is about four times larger than the probe pulse. For small fluences, the Dy lattice contracts within less than 30 ps given by the timescale of sound propagation through 18 nm Y and 80 nm Dy. In contrast, for high fluences, we first see a rapid Dy lattice expansion that relaxes on a few-nanosecond timescale. To visualize this threshold behavior, Fig. 3(b) shows the lattice strain ε(t=45 ps) as a function of the fluence. Below *F*_th_ = 2 mJ/cm^2^, a higher fluence leads to a more thorough disordering of the spin system and a concomittant lattice contraction. Above this threshold, additional energy leads to a linear expansion indicative of preferential excitation of phonons. For lower starting temperatures, a higher threshold fluence is required. The excitation of the spin system saturates due to the abrupt decrease of the specific heat of the spin system at *T_N_*.

Fig. 3(c) shows the temperature dependence of the photoinduced Dy strain for a fixed fluence of 6.8 mJ/cm^2^. For $T_{i}>T_{N}$, we observe the expected rapid phonon heat driven expansion superimposed with oscillations originating from coherent acoustic phonons (reflection of the strain waves at the interfaces of the multilayer) followed by a slow recovery of the lattice constant as heat flows towards the substrate. The red dashed line indicates the excellent agreement of a heat transport simulation according to the Fourier heat law through the layered system which we map on the strain axis by reading the experimentally determined strain in Fig. 1(a) at the calculated average temperature. For the heat diffusion we used the specific heat^40^ *C_p_* = 162.3 J/kg K and the heat conductivity^41^ *κ* = 11.7 W/mK reported in the literature (for details see Ref. 42). We note that the negative slope of the data from 0.1 to 1 ns (Fig. 3(c)) is exactly the same for all initial temperatures (gray dashed lines). Since for *T_i_* = 200 K this slope is dictated only by the phonon heat transport, we conclude that this is the case for all temperatures. The blue dashed line shows the strain resulting from a heat transport simulation for *T*_i_ = 100 K. The lattice contraction resulting from the increased spin excitation is captured by the simulations, since it is encoded in the negative thermal expansion coefficient. However, in contrast to the observed minimum around 500 ps, the simulation predicts a contracted lattice right after the excitation, because solving the Fourier-law for heat diffusion assumes the material parameters for thermal equilibrium, i.e., where the phonons and the magnetic system have the same temperature.

The Bragg peak width change (Fig. 3(d)) shows that in both the paramagnetic and the antiferromagnetic phases the width starts rising about 1 ps after the excitation. A simple masses and springs model^43^ confirms that the quasi-instantaneous stress originating from phonon heating yields a strong expansion wave with an exponential spatial profile starting at the surface. This wave travels into the Dy layer, and the maximum peak width is reached after about 18 ps when half the Dy layer is expanded. The width returns to near the initial value after 35 ps, when this strain front traverses the Dy-Y interface. This is consistent with the observation that at this time the average lattice constant determined from the peak shift (Fig. 3(c)) of Dy experiences a rapid contraction, as the expansion wave is transmitted towards the substrate. It is relevant to argue why the average lattice constant does not change in the first 35 ps for start temperatures between 80 and 130 K, although the peak width very strikingly shows an immediate inhomogeneous lattice deformation. The strong average expansion of Dy triggered by phonon heating must be cancelled by substantial contractive wavefronts starting at the Dy-Y interfaces due to the quasi-instantaneous loss of the magnetic order and the strong magnetostrictive coupling. The average cancellation suggests that at *t* = 35 ps about 25% of the energy heat up the magnetic system, while less than 75% heat up the phonon system according to the two Grüneisen constants.

The data in Fig. 3(c) confirm the threshold behavior: The laser induced average lattice strain remains unchanged from *T_i_* = 80 to 130 K in agreement with the nearly temperature-independent Grüneisen constants. Then, the observed initial strain gradually increases up to *T*_i_ = 180 K, where it saturates.

### Data analysis in a two-thermal-energy-model

The description of the UXRD data clearly showed that assuming a thermal equilibrium between the spin and phonon system on the nanosecond timescale is incompatible with the observations. Therefore, we analyze the flow of thermal energy among the spin and phonon systems within a model, where $Q_{S}$ and $Q_{P}$ are a function of time and space. We explicitly avoid a microscopic simulation in order to emphasize which conclusion can be drawn from the data under the assumption that the lattice strain is proportional to the energies in the two subsystems according to Eq. (1). To simplify the analysis, we start at times *t* = 45 ps where we may safely neglect a separate contribution of electrons, assuming them to be in thermal equilibrium with the phonons since electron-phonon coupling times in metals are expected on the order of few picoseconds at maximum.^7,10^

As a first step in our analysis, we calibrate the total energy densities $Q_{\text{tot},i}$ deposited by the laser excitation. At *T*_i_ = 250 K, all available energy $Q_{\text{tot}}^{250K}$ heats up the lattice, leading to a temperature increase of about 90 K corresponding to ε=0.2%. According to ellipsometric measurements of the sample, the optical absorption varies by less than 5%, so the total amount of energy deposited is $Q_{\text{tot}}^{T}=Q_{\text{tot}}^{250K}$ for all temperatures.

In the following, we discuss the measured data in terms of a nonequilibrium “two-thermal-energies-model” (TTEM) that satisfies energy conservation (Eq. (2)) and the linear superposition of magnetostrictive and thermoelastic strains (Eq. (3))

$$Q_{\text{tot}}(t)=Q_{S}(t)+Q_{P}(t),$$ (2)

$$\varepsilon_{\text{tot}}(t)=\varepsilon_{S}(Q_{S}(t))+\varepsilon_{P}(Q_{P}(t)).$$ (3)

This approach circumvents problems with the definition of temperature in nonequilibrium situations, the lack of literature data for the spin-phonon coupling constant and the separated contributions of the spin and phonon system to the heat conductivities, which would be necessary to obtain predictions from the differential equations of a standard TTM.^1,32^ We assume that the excitation pulse instantaneously deposits the previously calibrated initial energy density $Q_{\text{tot},i}$ in the Y cap layer and the top of the Dy layer via the very fast coupling of hot electrons to phonons and spins.^7^ The deposited energy is rapidly divided into the initial phonon energy density $Q_{P,i}$ and the initial energy $Q_{S,i}$ in magnetic excitations. Each energy density (stress) manifests itself as a lattice strain $\varepsilon_{P,S}(Q_{P,S})$, which superimpose to yield the measured strain. For analyzing the time resolved data, we take into account the effect of in-plane clamping of the lattice expansion according to Poisson's ratio (dashed lines in Fig. 2) as detailed in Ref. 42. Since both Grüneisen parameters are approximately constant as a function of temperature (see Fig. 1(c)), a linear relation between imparted energy and lattice strain emerges. This makes the analysis applicable even though the spin- and the phonon system may not be internally thermalized to one temperature $T_{S,P}$ over the film depth.

**FIG. 4.**
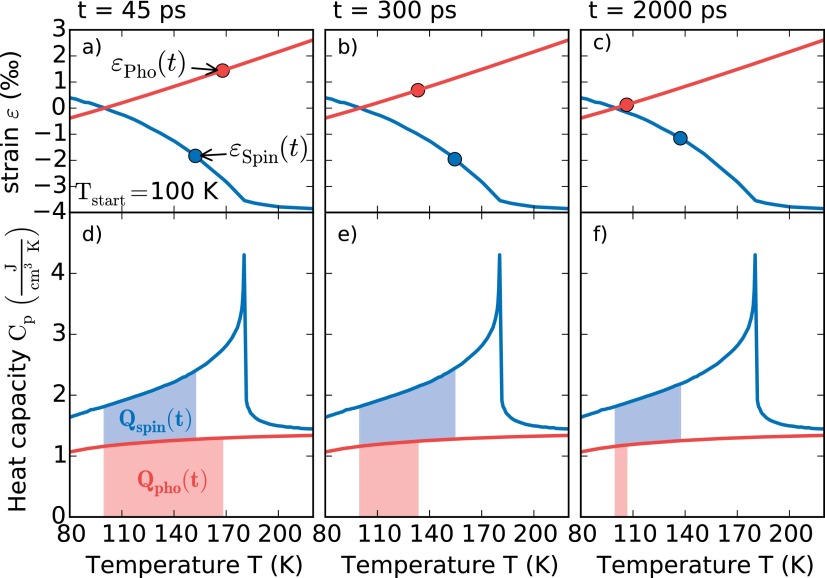
Visualization of the TTEM at *T_i_* = 100 K for three different time delays. The measured total strain $\varepsilon_{\text{tot}}(t)$ and the total heat energy *Q_tot_* allow deriving the individual phonon and spin contributions to the strain and the heat. The upper panels (a)–(c) show the separation of strain contributions according to Fig. 1(a) and the lower panels (d)–(f) show the transient heat as an area under the specific heat curve according to Fig. 1(c). Note that for the analysis only the energy densities are required. From the figures one can read that, e.g., after 2 ns, the *Q_S_* would correspond to an equilibrium spin temperature of 140 K whereas *Q_P_* corresponds to an equilibrium phonon temperature of 108 K. Although the temperature definitely has a strong gradient across the film, the analysis of the average heat *Q_P_*_;__*S*_ is robust, because of the linear relation $\varepsilon_{P;S}∼Q_{P;S}$.

First, we solve the system of equations, Eqs. (2) and (3), for the initial time *t_i_* = 45 ps, where the electron and phonon system are equilibrated but no energy has flown to the substrate. From the two measured variables $Q_{\text{tot}}(t_{i})$ and $\varepsilon_{\text{tot}}(t_{i})$, we calculate the energy in the spin $Q_{S}(t_{i})$ and the phonon system $Q_{P}(t_{i})$. This procedure is visualized in Figs. 4(a) and 4(d). The total energy density $Q_{\text{tot}}(t_{i})$ (red + blue area in Fig. 4(d)) in the sample is calibrated from the measurements in the paramagnetic phase, i.e., at $T_{i}=250\;K$ where the energy exclusively excites phonons, so that the deposited energy can therefore directly be found via Eq. (1) from the measured lattice strain ($\varepsilon_{\text{tot}}(t_{i})=\varepsilon_{P}(t_{i})$).

**FIG. 5.**
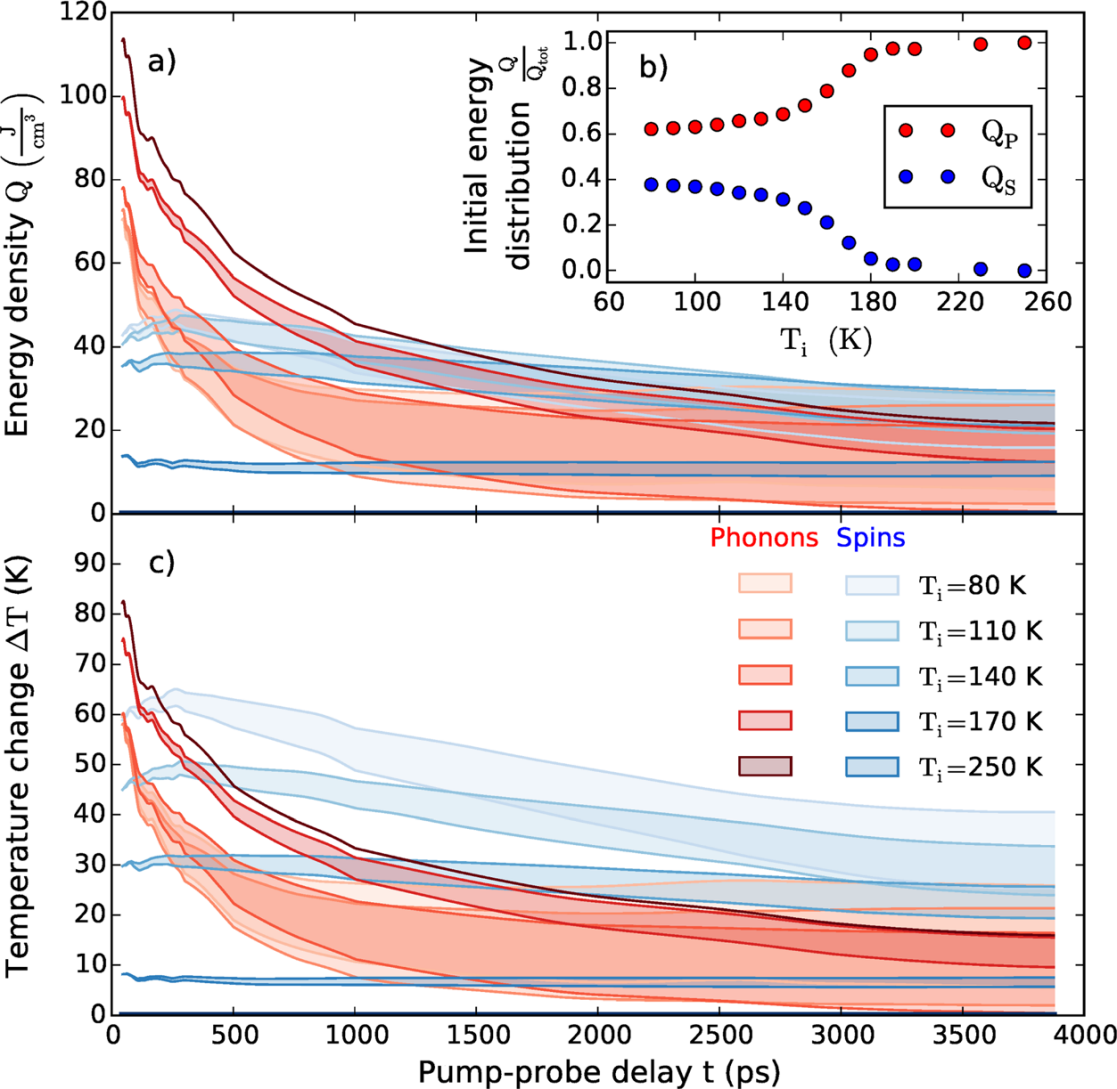
TTEM results extracted from the experimental data at a fluence of *F* = 6.8mJ/cm^2^. (a) Energy in the phonon- and spin excitations derived from the data as described in the text. $Q_{P}^{80K}$ and $Q_{P}^{110K}$ are hard to distinguish. (b) Initial energy distribution between phonons and spins at 45 ps. (c) Temperature changes obtained from (a) via the corresponding heat-capacities $C_{S,P}(T)$ (Fig. 1(b)). The resulting temperatures indicate a long-lasting non-equilibrium between phonons and spins. In panel (c), no temperature change of the spin system is plotted for $T_{i}=250\;K$ because its heat capacity is zero $C_{S}(250\;K)=0$.

For excitation with *F* = 6.8 mJ/cm^2^, this TTEM yields $Q_{S,i}$ and $Q_{P,i}$ and their fraction of the total energy is depicted in Fig. 5(b). It shows that essentially 35% of the energy is initially deposited in the spin system for $T_{i}\ll T_{N}$. Here, we assume that only the deposition of energy in each subsystem immediately leads to the stress according to the higher energy density, which causes the corresponding strain at delays larger than the time needed to the sound wave to propagate through Dy. We can safely assume that no energy has left the Dy layer by thermal transport shortly after excitation.

To obtain a solution of the system of equations for arbitrary times *t* as visualized in Fig. 4, we make two alternative approximations for the evolution of $Q_{\text{tot}}(t)$ in the dysprosium layer. $Q_{\text{tot}}(t)$ is reduced by phononic and electronic heat conduction to the substrate. As a lower boundary, we assume that the total heat flows out of the film as fast as if all energy would be stored in the phonons, i.e., $Q_{\text{tot}}^{T}(t)=Q_{\text{tot}}^{250K}(t)$. The direct flow of excitations from the magnetic system in the Dy layer to the adjacent nonmagnetic materials is not possible so that coupling energy in the spin system to phonons is the only channel of magnetic energy dissipation. Therefore, this assumption overestimates the heat flow to the substrate when a substantial fraction of $Q_{\text{tot}}^{T}(t)$ is in spin excitations and we obtain the lower curves in Fig. 5(a). The data analysis within the first approximation indicates that close to $T_{N}$ the energy in the spin system dissipates very slowly. To estimate an upper bound (Fig. 5(a)) we use the second approximation $Q_{\text{tot}}^{T}(t)=Q_{S,i}^{T}+Q_{P,i}^{T}(Q_{\text{tot}}^{250K}(t)/Q_{\text{tot},i}^{250K})$. It assumes that only the fraction of energy initially deposited in the phonon system can flow out of the film. This clearly overestimates the total energy in the Dy layer for long times *t* after the excitation, because the energy initially deposited in the magnetic system cannot leave the Dy layer and $Q_{\text{tot}}$ can never drop below $Q_{S,i}$. This approximation is especially suitable for initial conditions where $Q_{S,i}\ll Q_{P,i}$, i.e., at high temperatures. Fig. 5(a) shows the result of the TTEM data analysis for five representative temperatures using both approximations for $Q_{\text{tot}}(t)$. The heat dissipation from the phonon system is much faster than for the spin system. At very early times some transfer of thermal energy from the phonons into the spin system is observed. Referring to Fig. 4, we can observe that the main findings in Fig. 5 are already seen in the raw data. The upper panel of Fig. 4 illustrates that transient heat in the spin system leads to a contraction and transient heat in the phonon system leads to an expansion. The Grüneisen coefficients tell us that the energy in the spin system is three times more effective in straining the lattice than the energy contained in phonons. For the illustrated initial temperature of *T_i_* = 100 K, we observe at 45 ps that about three times more energy is required in the phonon system to nearly balance the contraction resulting from energy in the spin excitations. $\epsilon_{p}(45\;\text{ps})$ is slightly smaller than $\epsilon_{s}(45\;\text{ps})$, consistent with the slight contraction seen in Fig. 3(c). Around 300 ps, the phonon energy *Q_p_* has greatly decreased (Fig. 4(e)) by conduction of heat to the substrate, while $Q_{s}(300\;\text{ps})∼Q_{s}(45\;\text{ps})$. Therefore, the contractive strain *ϵ_s_* prevails and leads to the pronounced contraction seen in Fig. 3(c) around 300 ps. At 2 ns, there is almost no positive transient strain *ϵ_p_* due to phonons left (Fig. 4(c)) because *Q_p_* ∼ 0, and also *Q_s_* is greatly reduced (see Fig. 4(f)). In the raw data, this is seen as the recovery of the negative strain, which slowly approaches zero on this nanosecond timescale.

## DISCUSSION

Our analysis derives the flow of heat among the spin and lattice system directly from experimental data. In order to make it comparable to a conventional TTM, we present time-resolved temperature changes derived from the above TTEM using the static temperature-dependent heat capacity in Fig. 1(c). Initially, the temperature in the phonon system is higher than the temperature of the spin system, especially when *T*_i_ approaches *T*_N._ While the phonons in Dy cool rapidly to the substrate, *T*_S_ rises slightly within the first 200 ps even when the average temperature of the spin system is already higher than the average phonon temperature. Such behavior may be rationalized by a spatial nonequilibrium or—equivalently—by a nonequilibrium population of modes in microscopic theories. The subsequent cooling of the spin-system is found to be much slower. When the initial temperature $T_{i}$ approaches $T_{N}$ from below, the timescale for reordering (i.e., cooling) of the magnetic system dramatically increases: essentially, a decoupling of the two heat reservoirs from each other is observed. For example, for a starting temperature *T*_i_ = 170 K, Δ*T*_S_ ≃ 8 K remains constant for 4 ns although initially Δ*T*_P_ ≃ 70 K. From the experimental findings, we can give at least three indications that not only the two “temperatures” $T_{S}$ and $T_{P}$ are different, but that each of the subsystems is in a non-equilibrium situation. (i) As discussed above, the heat flow between spins and phonons is not controlled by the temperature difference. (ii) The heat simulation for *T*_i_ = 250 K which matches the experimental data predicts a strong temperature gradient in the Dy layer. Definitely, the phonons are spatially not in equilibrium over the sample thickness of 80 nm for more than 1 ns. (iii) The detected increase in the peak width in the first 35 ps while no peak shift is observed in the anti-ferromagnetic phase, which clearly calls for different spatial profiles of the excitations in the phonon and spin system. Probably, in the laser-heated region of the Dy layer $T_{P}>T_{S}$ while at the back interface to Y $T_{P}<T_{S}$. Potentially, the local excited mode spectrum cannot be approximated by a Bose-distribution with two temperatures at all.

## CONCLUSION

In conclusion, we have discussed UXRD data in the rare earth Dy to obtain clear evidence that the phonon and spin systems are mutually not in thermal equilibrium for several nanoseconds after optical excitation. We demonstrate a method to extract the transient energy densities $Q_{S}(t)$ and $Q_{P}(t)$ deposited in the spins and the phonons from the UXRD data. In the antiferromagnetic phase, approximately 35% of the energy heats up and disorders the spin system, leading to a rapid contraction of the lattice. Although the spins can exclusively dissipate their heat to phonons and electrons in Dy, the cooling rate of the magnetic system that finally leads to a reordering of the spin-system depends only weakly on the transient phonon temperature.

We hope that our experimental analysis will inspire theoreticians to model the spatiotemporal coupled excitations in rare earth metals and we believe that it is an important contribution in the context of the debate about ultrafast angular-momentum transfer to the lattice. Our observations are indicative for a rapid disorder in the spin system—inducing a contractive force, indicating a very strong spin-lattice interaction in the ordered phase. On the other hand the transfer of energy from the magnetic system to the phonons is very slow (on the nanosecond timescale), in particular, close to the phase-transition, giving strong evidence for a critical behavior. This suggests a rather weak spin-phonon coupling for (partially) disordered spins.
